# Involvement of Pruritus, Gut Dysbiosis and Histamine-Producing Bacteria in Paraneoplastic Syndromes

**DOI:** 10.3390/biomedicines13051036

**Published:** 2025-04-25

**Authors:** Doina Georgescu, Daniel Lighezan, Mihai Ionita, Paul Ciubotaru, Gabriel Cozma, Alexandra Faur, Ioana Suceava, Oana Elena Ancusa, Roxana Buzas

**Affiliations:** 1 Department V of Internal Medicine I, Center for Advanced Research in Cardiovascular Pathology and Hemostaseology, “V Babes” University of Medicine and Pharmacy, 300041 Timisoara, Romania; doina.georgescu@umft.ro (D.G.); dlighezan@umft.ro (D.L.); paul.ciubotaru@umft.ro (P.C.); suceava.ioana@umft.ro (I.S.); ancusa.oana@umft.ro (O.E.A.); buzas.dana@umft.ro (R.B.); 2 Department IX of General and Thoracic Surgery, Research Center of Thoracic Surgery, “V Babes” University of Medicine and Pharmacy, 300041 Timisoara, Romania; gabriel.cozma@umft.ro; 3 Department I of Anatomy and Embriology, “V Babes” University of Medicine and Pharmacy, 300041 Timisoara, Romania; faur.alexandra@umft.ro

**Keywords:** pruritus, paraneoplastic syndromes, gut dysbiosis, histamine-producing bacteria

## Abstract

**Background/Objectives:** Paraneoplastic syndromes (PNS), characterized by a large diversity of symptoms, may sometimes be the first clinical feature of a severe underlying disorder such as cancer. **Methods:** We report the case of a middle-aged male patient with no significant previous medical history, a nonsmoker or alcohol heavy drinker, complaining about generalized, recently onset itch. Given no reasonable explanation of pruritus after dermatological consultation and the unsatisfactory response to treatment, the patient was referred to gastroenterology with the suspicion of a cholestatic liver disease. **Results:** The abdominal ultrasound examination revealed gallstones and no dilation of the biliary tree. Numerous tests were run and came out negative, except for the slight elevation of C-reactive protein, mild dyslipidemia, and positivity for *H. pylori* antigen. The gut microbiota displayed important dysbiosis with a significant increase in the histamine-producing bacteria. Given this chronic pruritus became suspicious, thorax and abdominal CT were recommended and performed soon after. A large right mid-thoracic tumor image was found. Bronchoscopy came out negative for a tumor. After the CT-guided biopsy, the tumor turned out not to be a lymphoma, but a non-small cell lung carcinoma (NSCLC). **Conclusions**: Chronic pruritus was not associated with cholestasis in a patient with gallstone disease, but rather with a PNS, as the first clinical manifestation of NSCLC, triggering many diagnostic and therapeutic challenges.

## 1. Introduction

Paraneoplastic syndromes (PNS) are rare disorders characterized by a large diversity of symptoms that may sometimes be the first clinical feature of a severe underlying disorder such as cancer. From the clinician’s point of view, PNS are in fact a broad variety of symptoms that might feature a multitude of pathologies such as cutaneous, hematologic, endocrine, neurologic, musculoskeletal, cardiovascular, gastrointestinal and renal conditions. They are considered non-metastatic, systemic, distant effects of the neoplastic diseases that might be triggered by a particular immune reaction in response to cancer development, or by various substances produced by the tumor [1].

Although PNS may accompany at some point any kind of malignancy, given the rarity of those entities, the exact incidence and prevalence are still not known. According to epidemiological data, neurological PNS seem to be the most frequently described in clinical practice, male and female patients being equally affected [2,3].

Pruritus lasting more than 6 weeks, usually treatment nonresponsive, may be suspicious for a PNS. To consider pruritus a paraneoplastic condition in patients already diagnosed with a malignancy, one should rule out secondary, local presence of tumoral cells and side effects of cancer therapy [4]. Some studies have reported that pruritus of unknown etiology might be, in some cases, linked to oncological diseases, most of them being hematological conditions such as lymphoma, multiple myeloma and leukemia.

Pruritus as a PNS is rarely seen in solid tumors. However, some data suggest that pruritus was present in patients before the diagnosis of solid tumors like pulmonary, stomach, or laryngeal cancer. Pruritus was seldom observed in patients with breast and lung cancer [5]. Lung cancer is one of the most “productive” malignancies when it comes to PNS. About 10% of patients diagnosed with lung cancer may experience one or more types of PNS. Function of the histology of the lung tumor, the most often reported PNS are the endocrine syndromes such as inappropriate antidiuretic hormone secretion, Cushing syndrome due to ectopic adrenocorticotropic hormone, humoral hypercalcemia, as well as clubbing and hypertrophic pulmonary osteoarthropathy and Trousseau syndrome secondary to hypercoagulability. The presence of PNS is usually associated with a poor prognosis [6].

## 2. Case Report

**Figure 1:**
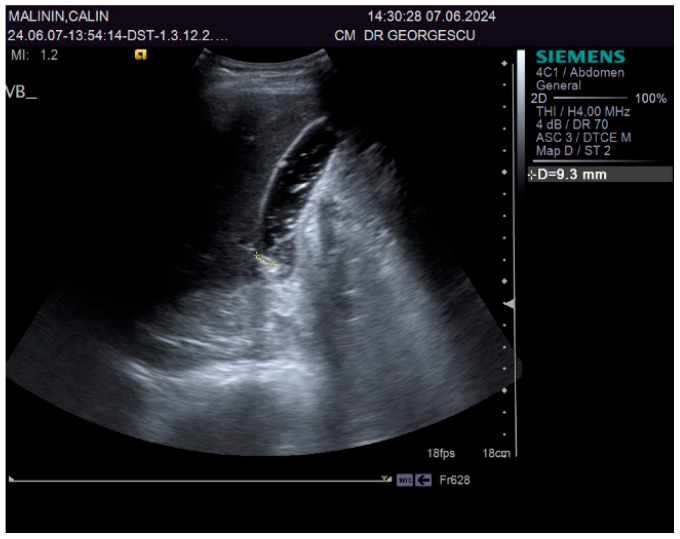
Ultrasound aspect: gallstones.

**Figure 2:**
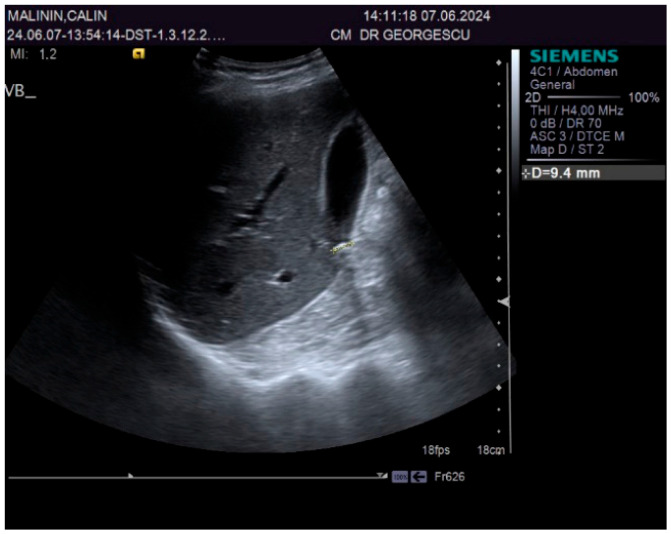
Ultrasound aspect: diffuse liver steatosis.

A 55-year-old male patient, complaining of recently onset, persistent generalized itching, was first examined by a dermatologist in the middle of May 2024 and no physical signs of skin disorder were found. Routine blood and stool workups were performed. No food or respiratory allergies were identified, and stool samples were negative for parasites. The recommended antihistamines were only partially effective, pruritus being present even during nights, resulting in a lowering of the patient’s quality of life. Given the mild dyspeptic-associated complaints, the patient was referred to a gastroenterological consultation on 7 June 2024 with a suspected biliary disorder. Medical history revealed no significant familial or personal pathologies, no smoking or alcohol drinking and no exposure to toxic substances. Physical examination found no particular pathological features, no peripheral palpable lymph nodes or skin lesions, normal respiratory and cardiovascular aspects, and blood pressure = 120/70 mmHg. However, the patient was overweight (BMI = 28 kg/m^2^) and displayed a mild palpatory tenderness of the middle and upper-right abdominal quadrant. Abdominal duplex ultrasound revealed multiple small echogenic formations adjacent to the gallbladder wall, likely cholesterol polyps. As illustrated in Figure 1 and Figure 2, two small calculi were also visualized in the infundibular zone. No biliary tree dilation was observed. Additional findings included mild liver steatosis, thickening of the stomach and duodenal walls, and a hyperechoic pancreatic texture. Aortic ectasia (2.7 cm in diameter) was noted just proximal to the distal bifurcation. No ascites or retroperitoneal lymph nodes were present.

As seen in Table 1 and Table 2, the patient underwent lab workups, including gut microbiota assessment. Numerous workups were within normal range, as illustrated in Table 1. However, a minor increase of total cholesterol (228 mg%) and LDL cholesterol (131 mg%), *H. pylori* antigen positivity (1.7 U), mild elevation of the inflammatory blood markers CRP (1.7 mg/dL) and fibrinogen (550 mg%), as well as severe decrease of diamine oxidase’s activity (DAO), were noticed (Table 1).

The particularities of the gut microbiota analysis performed using next-generation sequencing are displayed in Table 2.

As seen in Table 2, the study of the gut microbiota revealed important dysbiosis. Alteration of the bioindicators with a decrease in the H index of alpha biodiversity (Shannon index) = 2.1, modification of the F/B ratio = 0.7, particularities of the enterotype with *Bacteroides* spp. dominant aspects were observed. Alteration of the metabolic and functional microbiota with a decrease in mucosa-protective bacteria and a significant elevation of the histamine-producing bacteria were also noted.

**Figure 3:**
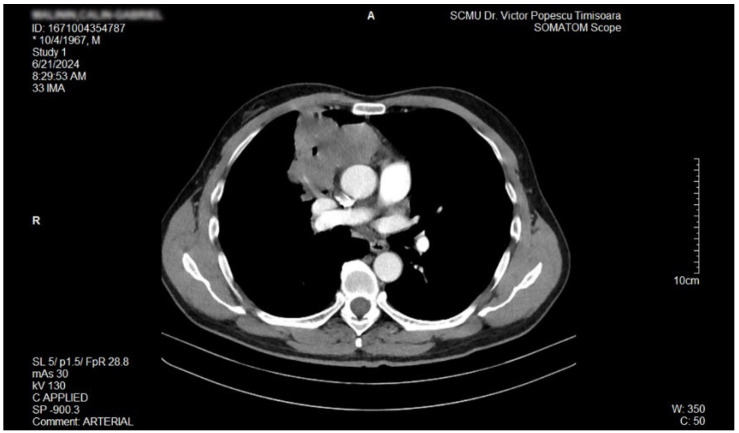
Contrast thorax CT: large right midthoracic tumor with mediastinal extension.(axial view.

**Figure 4:**
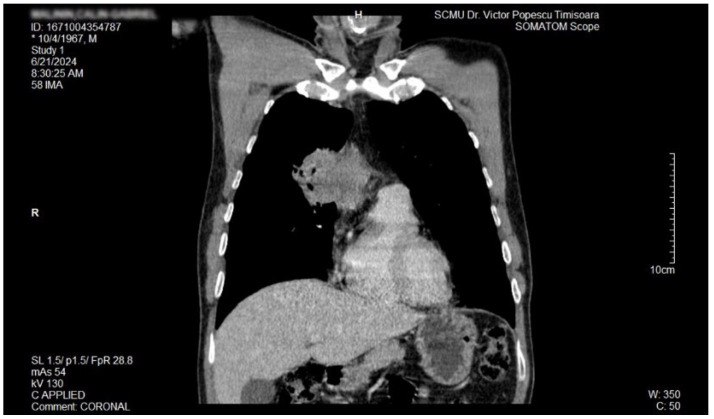
Contrast thorax CT: large right midthoracic tumor with mediastinal extension (coronal view).

**Figure 5:**
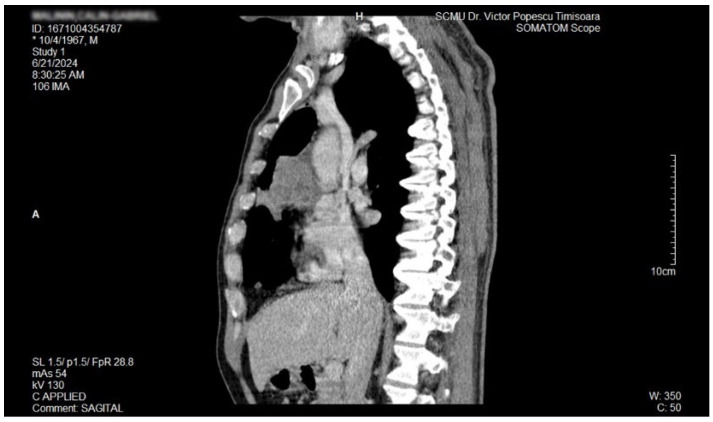
Contrast thorax CT: large right midthoracic tumor with mediastinal extension (sagittal view).

The proposal for *H. pylori* eradication treatment was initially postponed by the patient. However, the patient received symbiotics in order to alleviate the gut microbiota dysbiosis and also DAO supplementation, aiming at reducing the systemic effects of bacterial histamine overproduction. Further imaging studies were, however, ordered in the context of persistent, treatment-resistant pruritus that lasted for more than 6 weeks and eventually became suspicious for a malignancy. So that thorax and abdomen computed tomography (CT) were recommended and performed soon after, on 21 June 2024. As depicted in Figure 3, Figure 4 and Figure 5, a large right mid-thoracic tumoral formation with mediastinal extension of 7.8 cm/5 cm and a satellite lymph node of 7 mm placed in the anterior aspect of the upper right pulmonary lobe were discovered. The image was considered highly suspicious of lymphoma or pulmonary malignancy.

Bronchoscopy performed on 26 June 2024 came out negative: no mucosal pathological features were noted, so the suspicion of lymphoma still stood.

**Figure 6:**
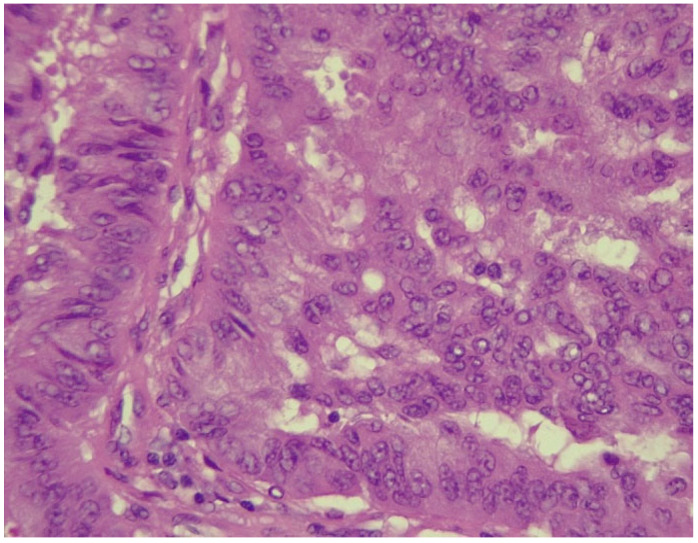
NSCLC showing tumor cells with marked pleomorphism and enlarged hyperchromatic nuclei (HE stained section, photographed using Leica DM750 microscope (Leica Microsystems, Heerbrugg, Switzerland) with digital camera objective at ×400 magnification).

**Figure 7:**
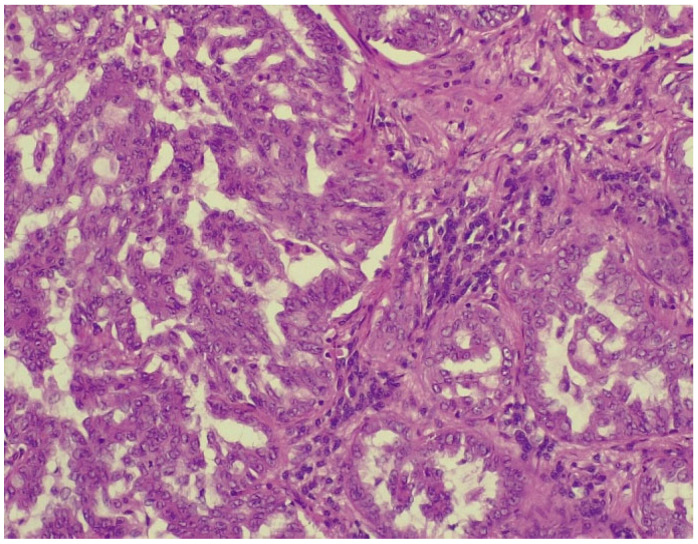
Alveolar spaces lined with pleomorphic cells (HE stained section photographed using Leica DM750 microscope (Leica Microsystems, Heerbrugg, Switzerland) with digital camera objective at ×200 magnification).

However, after CT-guided biopsy performed on 28 June 2024 and consecutive pathological sample examination, the suspicion of lymphoma was completely dismissed. The tissue sample analysis consisted of usual hematoxylin-eosin (HE) staining and immunohistochemistry (IHC). As depicted in Figure 6 and Figure 7, the optical microscopy examination of the tissue revealed aspects consistent with the diagnosis of a non-small cell lung carcinoma (NSCLC). The main microscopic description was of a non-mucinous type of lung adenocarcinoma.

**Figure 8:**
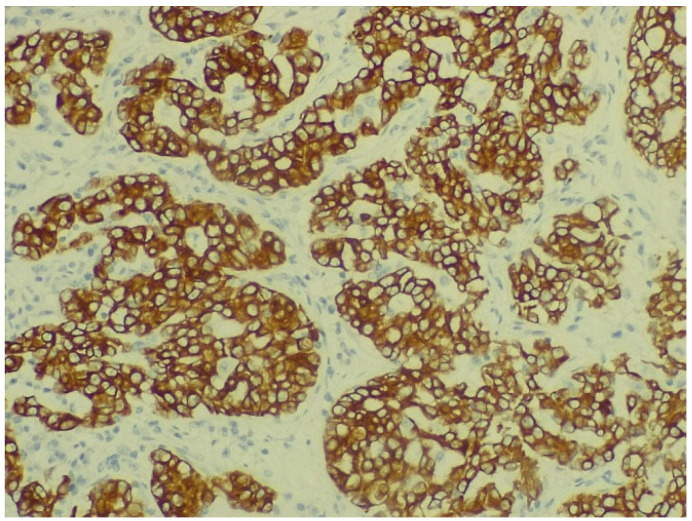
Immunohistochemical staining for cytokeratin 7 of non-mucinous type lung adenocarcinoma (objective ×400, Leica DM750 microscope (Leica Microsystems, Heerbrugg, Switzerland) with digital camera).

**Figure 9:**
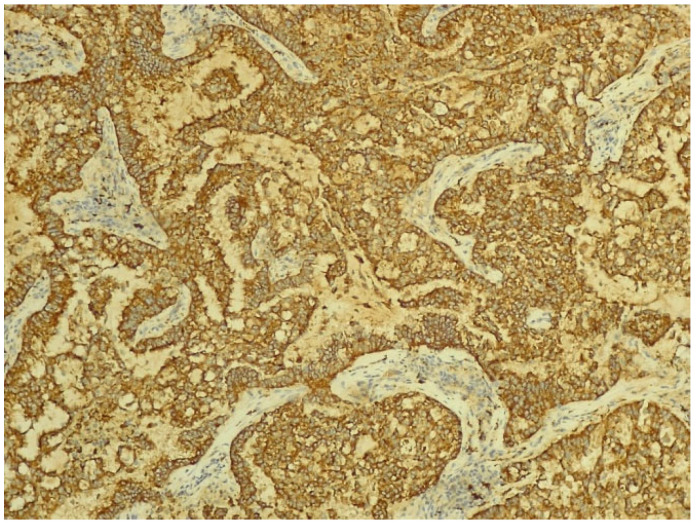
Immunohistochemical staining for napsin A of non-mucinous type lung adenocarcinoma (objective ×200, Leica DM750 microscope (Leica Microsystems, Heerbrugg, Switzerland) with digital camera).

The IHC of the tumoral cells of the non-mucinous type of lung adenocarcinoma showed positive results for markers cytokeratin 7 and napsin A (Figure 8 and Figure 9).

Additional studies of PD-L1 protein expression in the tumor were performed in a private laboratory, and the NSCLC showed a positive expression (Ventana, PD-L1, Roche Assay, Roche Diagnostics, Ventana Medical System, Inc., Tucson, AZ, USA). Molecular biology study performed using the next-generation sequencing method (Miseq Illumina Inc., San Diego, CA, USA) detected no mutations on the EGFR gene and no driver fusions at the level of ALK, ROS-1, NTRK, and RET.

At diagnosis, the NSCLC was staged as IIIA. Prior to initiating oncological treatment, the patient began *H. pylori* eradication therapy on 7 August 2024 (triple therapy: proton pump inhibitors and two antibiotics).

The patient underwent five cycles of combined chemo and immunotherapy—Carboplatin, Pembrolizumab, and Pemetrexed—which were well-tolerated. The pruritus rapidly decreased in intensity and totally disappeared soon after the first course of oncological treatment, clearly confirming that it was a paraneoplastic condition.

On 30 October 2024, the patient underwent a successful da Vinci robot-assisted right pulmonary bilobectomy. The pathological study of the resected tissue came out negative for viable tumoral cells. The postoperative recovery was rapid with a favourable short-term outcome.

## 3. Discussions

The diagnostic process in oncologic pathologies is often challenging. Given the complexity of clinical presentation in PNS, it is not unusual to mistake some symptoms and signs for other conditions, resulting in delaying the oncological evaluations and underdiagnosing cancers. So that the recognizing of a PNS is crucial, as they may precede the clinical manifestations of the oncologic disorders by a long time. In this regard, PNS act as potential disease modifiers by changing the outcome and prognosis of patients [7]. Realizing that the itching was a PNS represented for us the most important diagnostic step that rapidly led to the discovery of its true cause, respectively, the pulmonary cancer.

As others reported, chronic pruritus is frequently seen in clinical practice, associated with mood and sleep disturbances, and results in lowering the quality of life, comparable to chronic pain conditions [8]. In accordance with that, our patient experienced episodes of anxiety, depression, irritability, and had important difficulties in having a good night’s sleep, secondary to this intractable pruritus.

According to the International Forum on the Study of Itch, based on clinical grounds, the pruritus is classified into three groups. Group 1 associates patients with skin diseases, group 2 refers to patients with no skin lesions, and group 3 is represented by those with chronic pruritus. Patients with chronic pruritus of unknown origin may be at risk for malignancies, so additional diagnostic tools, like CT or other advanced imaging studies, may be needed [9]. Chronic pruritus of unknown origin in patients with generalized pruritus is reported in 3.6% to 44.5% of the population, being more frequent in the elderly [10]. These patients seemed, however, to rarely develop hematologic and biliary tree malignancies, after being followed up for 5 years, as a population-based study has reported [11]. The generalized pruritus of unknown etiology, lasting more than 6 weeks, was considered suspicious for a PNS and, consequently, the presented patient performed thorax and abdominal CT imaging studies. However, neither lymphoma nor biliary tree malignancy were found. Despite the epidemiological studies reporting that pruritus is most frequently associated with malignancies of the hematopoietic system, liver, and skin, however, a pulmonary mass was discovered.

Over time, the underlying pathways responsible for chronic pruritus were extensively studied. For decades, histamine was the main mediator associated with pruritus. However, current knowledge related to pruritus pathophysiology emphasizes the great complexity of participants. So, many actors may play a role in the pruritus apparition. Inflammatory cytokines such as IL-31 produced by Th2 lymphocytes, immune reactive cells, various skin cells, not to mention both central and peripheral neuronal networks, where tachykinin, a neuropeptide released from mast cells, may bind to various neurokinin receptors (NK1R, NK2R, NK3R), might be involved in pruritus development [12,13,14,15].

As many studies conducted in animals and human participants have reported, the gut microbiota might play an important part both in providing the healthy status of the individuals and in the development of various pathological conditions, as well [16,17,18,19,20,21].

Particularities of the gut microbiota composition and physiology reflect on the host immune status and may promote inflammation as a first step to the development of various pathologies [22]. The intestinal microbiome could play a significant role in the “gut-skin axis”, which seems to have a crucial implication on immunoregulation and immunotolerance processes, via dendritic cells and regulatory T cells (Th1, Th2, Th17, and Treg) [23]. Gut dysbiosis and abundance of pathogenic bacteria may result in a decrease of functional bacteria that produce butyrate and propionate, and consequently, continuously promote the cycle of inflammation and dysbiosis. A decrease in mucosal protective bacteria and leaky gut may favor the bacterial translocation, resulting in trimethyl anhydride (TMA)–induced pruritus [24]. The same aspect was noted in the patient we have presented, which displayed a severe low range of mucosa-protective bacteria, respectively, a decreased amount of the *Akkermansia muciniphila* and *Faecalibacterium prausnitzii*. Studies conducted in patients with pruritus and common skin diseases have reported the association of gut dysbiosis and emphasized some correlations of certain bacteria, such as *Anaerotruncus* spp., *Bacteroides* spp., and *Bacteroidaceae*, to the risk for the appearance of skin complaints [25]. Similarly, the patient in discussion exhibited important qualitative and quantitative alterations of the gut microbiota. He displayed a severe decrease in the Shannon index of the alpha biodiversity, alteration of the F/B ratio, and several modifications of the functionally and metabolically active gut bacteria. So that various LPS (+) bacteria, known to promote inflammation, were markedly increased.

The latest statistical data revealed that the burden of cancer is continuously increasing and has become a serious problem in global health management [26]. The relationship between gut microbiota dysbiosis and cancer has been vigorously studied over the past decades [27,28]. Recent studies emphasized the critical importance of the tumor microenvironment, which may interfere not only with cancer progression, but also with therapeutic response. Gut microbiota and tumor microenvironment are closely related via numerous pathways. It seems that gut microbiota may trigger the stimulator of interferon genes (STING)-type I IFN-dependent monocyte, reshaping the tumor microenvironment. Depending on the composition of the gut microbiota, the expression of the pattern recognition receptors on leukocytes and macrophages of the host immune cells may substantially vary. So that, microbiota may induce antitumorigenic IFN-based programs as a response to microbial STING agonists. A high-fiber diet may also promote type I IFN, resulting in the remodeling of the function of macrophages [29]. Interestingly, the patient presented in this case report displayed an imbalanced microbiota, with expression of type 1 of the enterotype, characterized by a *Bacteroides* spp.-dominant microbiome, as a result of a diet rich in animal protein and saturated fat and low in vegetal fibers.

Histamine-producing gut bacteria and a decrease in DAO activity may trigger various conditions, not only skin disorders [30]. The patient from this case report exhibited not only increased histamine-producing species such as *E. coli*, *Seratia* spp., *Klebsiella* spp., and *Enterobacter* spp., but also a significant decrease in serum DAO activity. These aspects qualified him for customized probiotic therapy and external supplementation of DAO. However, the implication of histamine seems to transcend the immune-allergic mediated conditions such as asthma, headache, muscular, abdominal and articular pain, as well as various skin conditions [31]. As recent data suggest, there is growing evidence about possible interferences of histamine with cancer pathophysiology. Histamine may play a role in cancer biology, not only in its initiation but also in its spreading, through interactions with particular cellular histamine receptors, where H_4_ plays a pivotal role [32,33,34]. In the future, these data may also serve to health providers as a starting point for the production of biomarker kits, in order to help clinicians to predict the prognosis of several cancers, by simply demonstrating the level of expression of various histamine receptor subtypes.

## 4. Conclusions

This is a case report of a middle-aged male patient with no significant medical history, complaining about generalized, recently onset itching, initially diagnosed with GSD and severe gut dysbiosis with an important increase in the histamine-producing bacteria. The intractable pruritus became suspicious for a PNS, posing many diagnostic and therapeutic challenges. A NSCLC, with no specific signs of a pulmonary condition, was eventually confirmed and consecutively treated by chemo and immunotherapy and robotic-assisted surgery, with a good short-term outcome.

## Figures and Tables

**Table 1:** Biologic baseline parameters.

Parameters	Baseline Data	Normal Range
Hb	13.1 g/L	12–15 g/L
L	7.5 × 10^3^/mm^3^	4–11 × 10^3^ mm^3^
Eo	0.3	0.04–0.40
Plt	258 × 10^3^/dL	150 × 10^3^–450 × 10^3^
ALT	31 u/L	7–56 u/L
AST	28 u/L	10–40 u/L
GGT	32 iu/L	0–30 iu/L
ALP	25 iu/L	30–120 iu/L
TB	0.8 mg/dL	0.1–1.2 mg/dL
Glu	88 mg/dL	70–100 mg/dL
Hb A1c	5.4%	<5.7%
HOMA-IR	1.6	<2.5
T Chol	228 mg/dL	<200 mg/dL
LDL	131 mg/dL	<100 mg/dL
CRP	1.7 mg/dL	<0.5 mg/dL
Fibrinogen	550 mg/dL	200–400 mg/dL
Creat	0.9 mg/dL	0.7–1.3 mg/dL
TSH	3.5 mU/L	0.5–5 mU/L
Ig E	61 IU/mL	0–100 IU/mL
HBsAg	negative	negative
HCV Ab	negative	negative
HIV serology	negative	negative
AN Ab	negative	negative
AM Ab	negative	negative
p ANCA	negative	negative
Ce Ab	negative	negative
Breath test lactose/sorbitol	negative	negative
CEA	2.1 ng/mL	0–2.5 ng/mL
CA19-9	14.5 U/mL	<37 U/mL
DAO	2.8 U/mL	>10 U/mL
*H. pylori* Ag	1.7 U/mL	<0.7 U/mL
Stool NGS exam	dysbiosis	normobiosis

Legend: Hb = hemoglobin, L = leukocyte, Eo = eosinophils, Plt = platelets, ALT = alanine aminotransferases, AST = aspartate-aminotransferases, GGT = gamma GT, ALP = alkaline phosphatase, TB = total bilirubin, Glu = glucose, Hb A1c = glycosylated hemoglobin, HOMA-IR = Homeostatic Model Assessment for Insulin Resistance, T Chol = total cholesterol, LDL = low density of lipoproteins, CRP = C-reactive protein, TSH = thyroid stimulating hormone, Ig E = immunoglobulin E, HBs Ag = Hepatitis B surface antigen, HCV Ab = Hepatitis C antibodies, HIV = human immunodeficiency virus, AN Ab = antinuclear antibodies, AM Ab = anti mitochondrial antibodies, p ANCA = perinuclear anti neutrophil cytoplasmic antibodies, Ce Ab = celiac disease antibodies, CEA = carcino-embryonic antigen, CA19-9 = carbohydrate antigen, DAO = diamine oxidase NGS = next generation sequencing.

**Table 2:** Gut microbiota features.

Microbiota Alterations		Range
Shannon index		2.14
F/B ratio		0.7
LPS + bacteria	*Citrobacter* spp.	0.02
	*Providencia* spp.	0.003
	*Seratia* spp.	0.001
	*Suterella* spp.	8.042
H producing bacteria	*E. coli*	0.44
	*Seratia* spp.	0.002
	*Klebsiella* spp.	0.551
	*Enterobacter* spp.	0.005
Mucosa protective bacteria	*Akkermansia muciniphila*	0.006
	*Faecalibacterium prausnitzii*	1.02
Enterotype		1

Legend: F/B = *Firmicutes*/*Bacteroidetes*, LPS = lipopolysaccharides, H = histamine.

## Data Availability

Data will be provided upon written request.

## Abbreviations

The following abbreviations are used in this manuscript:

MDPI	Multidisciplinary Digital Publishing Institute
DOAJ	Directory of open access journals
TLA	Three-letter acronym
LD	Linear dichroism
